# The Performance Testing and Analysis of Common New Filter Materials: A Case of Four Filter Materials

**DOI:** 10.3390/ma17122802

**Published:** 2024-06-08

**Authors:** Fenggang Sun, Xin Zhang, Tao Xue, Ping Cheng, Tao Yu

**Affiliations:** 1 School of Resources Engineering, Xi′an University of Architecture and Technology, Xi’an 710055, China; xinred31711@xauat.edu.cn (F.S.); xt03230151@xauat.edu.cn (T.X.); chengp_314@163.com (P.C.); 2 Wuhan Second Ship Design and Research Institute, Wuhan 430205, China

**Keywords:** particles, filter materials, fiber structure, performance differences

## Abstract

The complex air environment makes it urgent to build good and safe indoor environments, and the study and application of new materials have become the focus of current research. In this study, we tested and analyzed the structural parameters and filtration performances of the four most commonly used new filter materials in the current market. The results showed that all four new filter materials showed a trend of first increasing and then decreasing their filtration efficiency with an increase in filtration velocity. The filtration efficiency of the materials was as follows: PTFE > glass fiber > nanomaterial > electret. The filtration efficiency of all materials reached its maximum when the filtration velocity was 0.2 m/s. The filtration efficiency of the PTFE for PM_10_, PM_2.5_, and PM_1.0_ was higher than that of the other three materials, with values of 0.87% to 24.93%, 1.21% to 18.69%, and 0.56% to 16.03%, respectively. PTFE was more effective in capturing particles smaller than 1.0 μm. Within the testing velocity range, the resistance of the filter materials was as follows: glass fiber > PTFE > electret > nanomaterial, and the resistance of the four materials showed a good fitting effect. It is also necessary to match the resistance with the filtration efficiency during use, as well as to study the effectiveness of filter materials in blocking microorganisms and absorbing toxic gases. Overall, PTFE showed the best comprehensive performance, as well as providing data support for the selection of related materials or the synthesis and research of filter materials in the future.

## 1. Introduction

Environmental pollution has long been a hot topic and cause for concern [1,2]. Small particles suspended in the air are one of the main sources of air pollution, and they can range in size from hundreds of nanometers to tens of micrometers [3]. People living in environments with high concentrations of pollutants for an extended period of time can suffer varying degrees of physical illness and even death [4]. After particles of different sizes enter the human body, they will remain or deposit in different positions, causing various problems [5,6,7,8]. For example, particles larger than 10.0 μm are more easily blocked by organs such as the nose and mouth [5]. Particles between 5 and 10 μm mainly stay in the upper respiratory tract (i.e., nose, pharynx, and throat) [6]. Particles of 2.5 μm can enter the bronchioles, alveolar sacs, and alveoli of the lungs, easily attacking the human respiratory system and causing serious harm to the bronchi and other tissues [7]. Particles smaller than 1.0 μm can directly enter the human respiratory system, attacking the alveoli, as well as the circulatory system, triggering various diseases (such as cardiovascular and respiratory diseases) and posing a serious threat to human health. Therefore, determining how to reduce the concentration of particulate matter in the air in order to create a safe and healthy living environment is one of the primary goals at present.

To date, a large amount of research has been conducted on the removal of pollutants from the air [9,10,11,12,13,14], such as filtration with non-woven materials [9], nanobubble encapsulation for dust reduction [10], water spraying for decontamination, and starting from the source [11], analyzing the sources and distribution of pollutants to reduce their generation [12], the methods for controlling the production of particle suspensions [13], and the selection and experimental evaluation of the intake filter materials for internal combustion engines [14]. However, based on the current overall technological maturity and practical applicability in application areas, along with a comprehensive comparison of several existing technologies, it can be concluded that fiber filtration technology is one of the most widely used technologies at present, among which, air filters are widely used due to their ability to filter and purify particulate matter in the air [15]. The most commonly used air filtration materials in the current market are mainly melt-blown ultrafine-fiber non-woven fabrics [16], but the scale of melt-blown fibers is mostly above micrometers, meaning that they cannot effectively intercept tiny, deadly particles in the air. Therefore, the preparation and optimization of fiber materials have always been among the main directions in research on high-performance filtration materials [17]. Domestic and foreign scholars have achieved certain results through extensive related research [18,19,20,21]. The focus of such research is on improving the efficiency of filter materials [18], as well as the development of new materials [19,20,21], and so on. However, due to the changing environment and increasing demand for the removal of small- and medium-sized particles from the air, research on new materials has always been the mainstream direction of current research.

In order to achieve the ultimate goal of a high efficiency and low resistance, existing scholars and research institutions have developed some filter materials over a considerable period of time and applied them in practical production and daily life. The most widely used new filter materials include electret materials [22], nanomaterials [23], glass fiber materials [24], and PTFE materials [25]. Electret air filtration materials not only have their original mechanical blocking effect, but they also rely on electrostatic force to directly attract charged particles in the air and capture them, or induce neutral particles in the air to generate polarity and then capture them. This is more effective in filtering submicron particles in the air, significantly improving filtration efficiency without increasing air resistance. The findings of many scholars have confirmed this point [26]. However, the electrostatic persistence of electret materials is one of the main factors limiting their performance [27]. Therefore, it is necessary to conduct long-term research on the persistence of electrostatic effects in electret materials and their changes in filtration performance over time. The relevant literature shows that, after a certain period of time [28], the surface charge of the electret material will decrease, and the electrostatic effect will weaken until mechanical filtration gradually replaces electrostatic filtration. Therefore, when the electret material has an electrostatic effect, its filtering effect is relatively good [29]. After the electrostatic effect disappears, the mechanism of action is the same as that of ordinary filter materials. Nanomaterials generally consist of some nanoscale particles that are processed into fibers or onto fabric filter materials through certain methods, resulting in a finer fiber diameter and a higher specific surface area, with some special functions. The relevant research and development have provided progress and performance improvements of different functional nanofiber air filtration materials [30], providing reference data for the development of functional materials. Glass fiber filter materials have a certain pore structure. When harmful particulate matter passes through the filter medium, it is adsorbed and deposited on the surface and inside the filter medium under mechanical or electrostatic action, achieving filtration [31]. The surface of PTFE filter materials is smooth and resistant to chemical substances. It is overlaid on the surface of ordinary filter materials to act as a one-time dust layer, trapping all dust on the surface of the membrane and achieving surface filtration [32]. This has unparalleled advantages over traditional filter materials. PTFE has the characteristics of a high peel strength, high air permeability, low resistance, and uniform pore size distribution.

In summary, it can be seen that all four filter materials exhibit an efficient and low-resistance filtration performance, and they have also been widely used due to their characteristics. However, they all have certain drawbacks, and they are still relatively vague in terms of their practical use, with differences in selection. As a result, there has been little comparison between the four filter materials mentioned above, especially in terms of their performance differences in filtering particulate matter, where they are slightly insufficient. In addition, due to the relatively large differences in the preparation concentration and cost of these materials, people tend to consider their cost-effectiveness comprehensively in practical use. This is because most existing filter materials are made of polyester or non-woven fabric materials, which have low raw material prices along with mature and simple preparation processes, so these are still the main materials used at present [33]. However, the existing preparation processes for these four materials have not yet been widely used, due to their relative complexity and high cost. For example, PTFE materials require surface coating [34], which increases the difficulty and cost of the material preparation process. With different uses and purposes in various places in practical use, filters are generally used continuously for more than 10 h [35]. However, after a period of use, filters may experience an increased resistance and decreased efficiency, so they must be replaced. In addition, sometimes, in order to welcome inspections and patrols, the filter will also be replaced at a certain time, so as to have a larger area of application. For this purpose, this study focuses on the performance differentiation of four existing new materials, in order to provide more suitable environment and usage conditions. It is more practical to provide preferred filter materials with relatively mature production processes when people demand larger and more frequently replaced filters in the post-pandemic era.

Therefore, this paper focuses on the above practical issues and presents in-depth research on the structural parameters and filtration performances of the four most commonly used new filter materials in the current market; it also provides data support for the selection of related materials or the synthesis and research of filter materials in the future.

## 2. Materials and Methods

### 2.1. Parameters

The filtration efficiency of air filters was calculated using Equation (1) [36]:

(1) $$\eta =\frac{C_{1}-C_{2}}{C_{1}}\times 100\%$$

where η is the filtration efficiency (%), *C*_1_ is the concentration of particulate matter before filtration (μg/m^3^), and *C*_2_ is the concentration of particulate matter after filtration (μg/m^3^).

The counting efficiency of air filters was calculated using Equation (2) [36]:

(2) $$\eta_{i}=(1-\frac{N_{2i}}{N_{1i}})\times 100\%$$

where $\eta_{i}$ is the counting efficiency (%), $N_{1i}$ is the average concentration of particles greater than or equal to a certain size before filtration (particles/L), and $N_{2i}$ is the average concentration of particles greater than or equal to a certain size after filtration (particles/L).

The filtration velocity was the same before and after the filters were applied, and the cross-sectional area was equal. The filtration resistance could be expressed by the static pressure difference. The filtration resistance was calculated using Equation (3) [36]:

Δ*P = P*_2_ − *P*_1_ (3)

where *P*_1_ is the static pressure before filtration (Pa) and *P*_2_ is the static pressure after filtration (Pa).

The filling rate cannot be directly determined. By measuring the density of the filter material and calculating its ratio to the density of the material used in the filter material, the filling rate was calculated using Equation (4) [37]:

(4) $$\alpha =\frac{\rho_{1}}{\rho_{2}}$$

where α is the filling rate (%), $\rho_{1}$ is the density of the filter layer (kg/m^3^), and $\rho_{2}$ is the density of the filter layer material (kg/m^3^). According to the definition of density, to determine the density, it is necessary to know the mass and volume of the sample.

### 2.2. Experimental Systems

**Figure 1:**
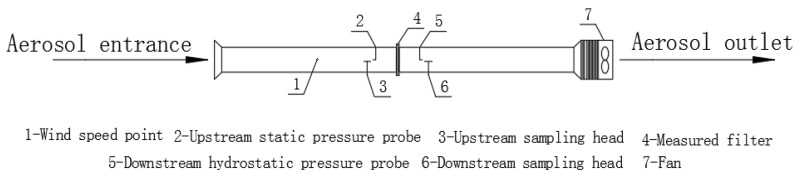
Experimental setup.

The experimental setup is shown in Figure 1. A GRIMM1.109 Portable Aerosol Spectrometer was used to measure the concentration of particles before and after the air filters were applied, and it was supplied by Beijing Saak-Mar Environmental Instrument Ltd., Beijing, China. The upper limit for the concentration measurement was 2,000,000 P/L, the measurement range was 0.1~100,000 μg/m^3^, and the repeatability was 5%. A HD2114P.0 Portable Micromanometer was used to measure the filtration resistance, and it was supplied by DeltaOHM Co., Ltd., Padova, Italy, with an accuracy of ±2% reading + 0.1 m/s. The pressure range was ±0.4% F.S. An HD37AB1347 Indoor Air Quality Monitor was used to measure the velocity, and it was supplied by DeltaOHM Co., Ltd., Italy, with an accuracy range of ±3%. A micrometer was used to measure the thickness, with an accuracy of 0.01 mm. A JSM-6510LV scanning electron microscope was used for analysis, and it was supplied by Japan Electronics Co., Ltd., Amagasaki, Japan; its magnification was 5~30 million times and its resolution was up to 3.0 nm. A TSI7525 Indoor Air Quality Meter was used to measure the temperature and humidity, and it was supplied by TSI Instrument Beijing Co., Ltd., Beijing, China. The temperature measurement range was 0~60 °C, with a measurement accuracy of ±0.6 °C and a 0.1 °C resolution. The relative humidity measurement range was 5~95% RH, the measurement accuracy was ±3% RH, and the resolution was 0.1% RH. The average concentrations over 5 min before and after the testing were used in the calculations to reduce experimental errors. The experimental steps were as follows: firstly, the material to be tested was installed in the middle of the pipeline and fixed. Then, the front and back ends of the filter were tested separately. Two groups of the front and back ends were tested for 5 min each, and the average concentration value of 5 min was taken as the calculation value to reduce experimental errors. After the final test, the test materials were replaced and the above experimental steps were repeated.

### 2.3. Experimental Materials

**Figure 2:**
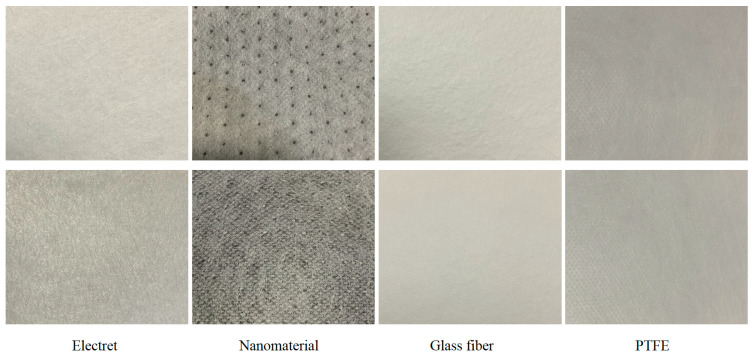
Physical images of filter materials.

Physical images of the filter materials are shown in Figure 2.

The relevant physical parameters of the four materials are shown in Table 1, as determined according to relevant tests and formula calculations of the physical objects.

## 3. Results and Discussion

### 3.1. Distribution of Atmospheric Particles

**Figure 3:**
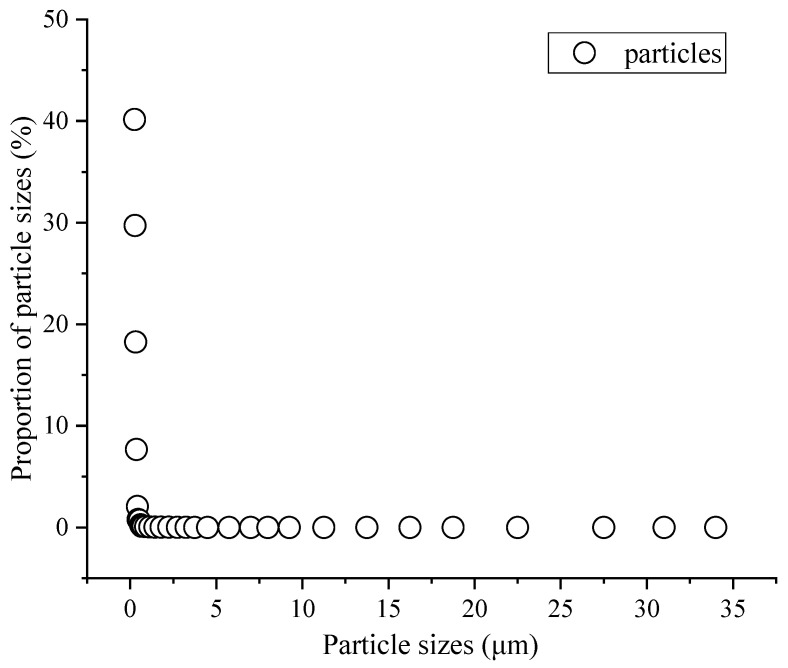
Distribution of atmospheric particles.

Outdoor atmospheric dust in Xi’an was used as the test dust source for the research presented in this paper [38], in order to reflect the true performances of these four materials in the current market, as well as to make their corresponding testing more practical. Figure 3 shows the particle size distribution of the atmospheric particles during the testing period of this experiment.

From the particle size distribution in Figure 3, it can be seen that smaller particles occupied the main position in the air. Particles smaller than 2.5 μm accounted for 99.97% of all particulate matter, while particles smaller than 1.0 μm accounted for the vast majority (~99.84%), of which particles smaller than 0.5 μm accounted for approximately 69.86%. This shows that small particles were the most abundant in the air of Xi’an, while larger particles were less abundant, due to gravity. This is consistent with the results presented in [39], which verify the findings of this paper; it also indicates that the size distribution of atmospheric particles in the testing area of Xi’an was mainly dominated by fine particles, which are more likely to enter the human body and cause varying degrees of harm, or even death. Therefore, people now pay more attention to the capture and purification of small- and medium-sized particles in the air compared to in the past. How to improve the capture effect of air filters is one of the key factors in evaluating filter performance.

### 3.2. The Microstructure of Filter Materials

**Figure 4:**
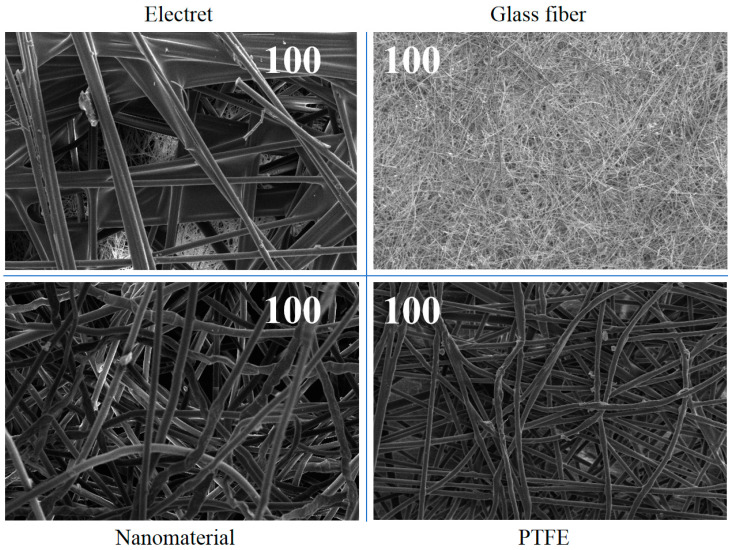
Scanning electron microscope images of the different materials (100 times).

Figure 4 shows scanning electron microscope images of the four materials.

From Figure 4, it can be seen that the surface of each fiber in the different materials was smooth, and the distribution between fibers presented a natural twisted state, with a smooth and orderly fiber surface. In addition, the pore size between fibers in each material varied, with different trapping effects on particles of different sizes. The larger the pore size, the easier it is for particles to pass through fibers, resulting in a decrease in the particle capture efficiency [40]; on the other hand, the smaller the pore size, the more particles cannot pass through the fibers and are captured by them, improving the fibers’ ability to capture particles. The structure between fibers in electret materials is relatively loose, and the porosity is relatively high. Although nanomaterials appear relatively tight between fibers, they are actually relatively loose, and the overall toughness between fibers is relatively poor. This is because nanomaterials are mostly processed by other materials on the surface of fibers; thus, compared to other natural materials, nanomaterials exhibit fractures or obvious processing states. Glass fiber and PTFE materials have tighter fibers, but due to their thickness, glass fiber materials exhibit multi-level combinations between them. PTFE materials exhibit an alternation between coarser and finer fibers, resulting in a distinct “raised” structure of the fibers in the filter material, also effectively regulating the distance between fibers, thereby adjusting the fiber filling density. This means that PTFE materials have an excellent ability to trap particles. It was carefully discovered that there were a large number of tiny “nodes” of different sizes distributed on the surface and middle of PTFE materials, which were crystalline molecules of PTFE that had not been stretched and unfolded. This is consistent with the literature, which verifies the correctness of this article [40]. The presence of “nodes” in the filter material strengthens the “raised” structure of the fibers, achieving further adjustment of the filling method and filling density between the PTFE fibers, and this helps to improve the filtration performance of the PTFE material. Through the microstructure, it could also be seen that the resistance could be reduced by changing the fibers’ own structure, without affecting the filtration efficiency. According to the relevant literature [19], fibers can be composed of porous media with a large specific surface area. When passing through dust sources, this can not only efficiently capture pollutants, but also provide multi-channel airflow, thereby reducing the resistance without affecting the filtration efficiency. For example, graphene materials [19] and carbon nanomaterials [41], etc., all show a good comprehensive performance.

### 3.3. Influence of Filtration Velocity

The filtration performances of four new filter materials under different filtration velocities were tested and analyzed according to relevant standards. The concentration distribution in the air during the testing process was as follows: the concentration range for PM_10_ was from 10.8 to 74.6 μg/m^3^, the range for PM_2.5_ was from 8.6 to 56.8 μg/m^3^, and the range for from PM_1.0_ was 5.1 to 35.2 μg/m^3^. Table 2 shows the trend of the filtration efficiency changes at different filtration velocities.

**Figure 5:**
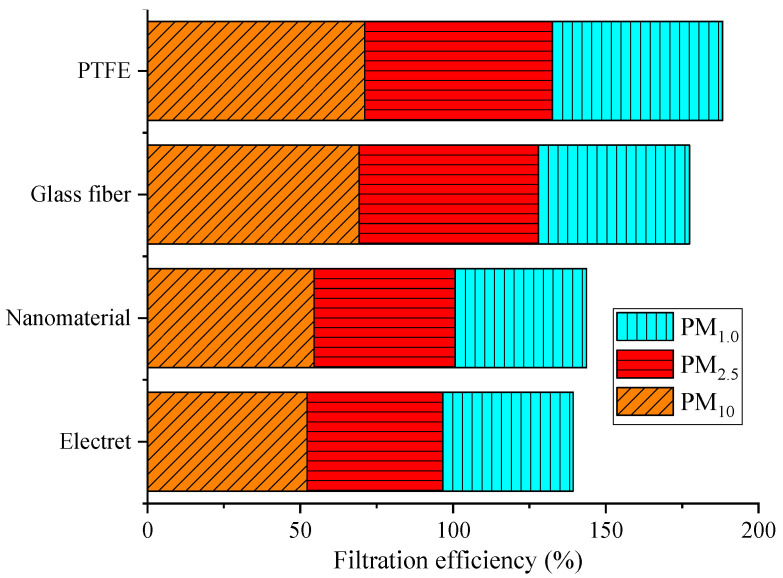
Differences in the PM filtration efficiency of different materials.

From Table 2, it can be seen that all four new filter materials exhibited a trend of first increasing and then decreasing their filtration efficiency with an increase in the filtration velocity. The filtration efficiency range of the electret material for PM_10_ was from 27.55% to 52.26%, for PM_2.5_ it was from 24.01% to 44.35%, and for PM_1.0_ it was from 22.48% to 42.69%. The filtration efficiency range of the nanomaterial for PM_10_ was from 28.57% to 54.52%, for PM_2.5_ it was from 28.32% to 46.17%, and the range for PM_1.0_ was from 26.18% to 43.02%. The filtration efficiency range of the glass fiber material for PM_10_ was from 43.95% to 69.30%, for PM_2.5_ it was from 34.92% to 58.56%, and for PM_1.0_ it was from 23.97% to 49.51%. The filtration efficiency range of the PTFE material for PM_10_ was from 41.25% to 71.10%, for PM_2.5_ it was from 31.61% to 61.43%, and for PM_1.0_ it was from 28.36% to 55.72%. The filtration efficiency of the materials was as follows: PTFE > glass fiber > nanomaterial > electret. The filtration efficiency of all materials reached its maximum when the filtration velocity was 0.2 m/s, and overall, the filtration efficiency of PTFE for PM_10_, PM_2.5_, and PM_1.0_ was higher than that of the other three materials, with ranges of 0.87% to 24.93%, 1.21% to 18.69%, and 0.56% to 16.03%, respectively. Figure 5 shows the differences in the PM filtration performance among the different filter materials at the optimal filtration velocity.

From Figure 5, it can be seen that, at the optimal filtration velocity, the filtration efficiency of PTFE for PM_10_ was 18.83% higher than that of the electret material, 16.57% higher than that of the nanomaterial, and 1.80% higher than that of the glass fiber material. The filtration efficiency of PTFE for PM_2.5_ was 17.08% higher than that of the electret material, 15.27% higher than that of the nanomaterial, and 2.87% higher than that of the glass fiber material. The filtration efficiency of PTFE for PM_1.0_ was 13.03% higher than that of the electret material, 12.70% higher than that of the nanomaterial, and 6.21% higher than that of the glass fiber material. This was because PTFE is a membrane-covered filter material [42]. When dusty gas passes through the filter material, most of the dust particles will be trapped on the surface of the PTFE material due to interception, and they will not directly enter the interior of the fibers. Therefore, filtration is carried out on the surface of the PTFE material, achieving surface filtration. In contrast, other filter materials experience a process of particles entering the interior of the fibers from the surface. Therefore, the surface filtration effect of PTFE was better under the same conditions. In addition, for small particles, Brownian motion plays a dominant role [43], and as shown in Table 1, the porosity of PTFE was smaller. At the same filtration velocity, due to the combined effects of inertia and interception, the probability of collision between particles and fibers increased, resulting in a significant increase in the capture efficiency of the PTFE material for PM_1.0_, PM_2.5_, and PM_10_. Overall, it can be seen that the new PTFE filter material on the market currently had a better performance, whereas that of the electret material was the lowest in relative terms; this was because the electrostatic effect on the surface of the electret weakened or disappeared at that time, meaning that only mechanical filtration took place [28], and its relatively large porosity made it unable to effectively capture particles, resulting in a relatively low efficiency. This conclusion is consistent with the literature, which verifies the findings of this paper [19].

### 3.4. Differences in Counting Filtration Efficiency for Different Particle Sizes

**Figure 6:**
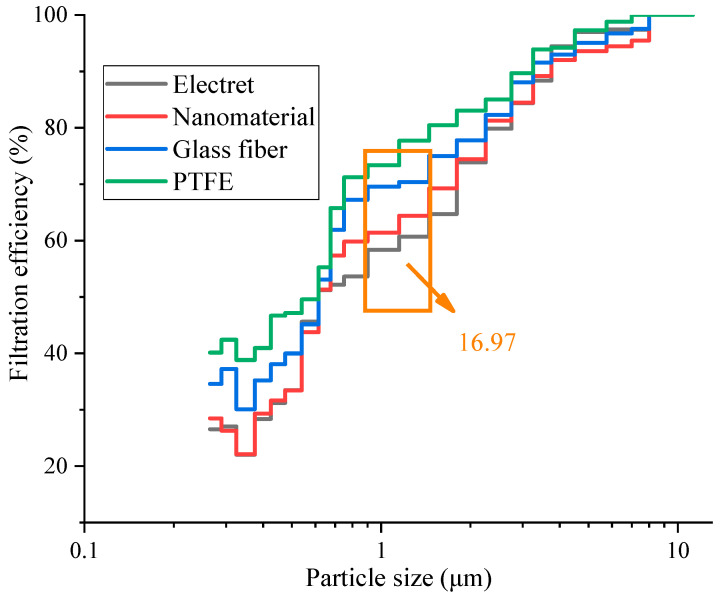
Counting filtration efficiency under different particle sizes.

The counting efficiency for different particle sizes under different materials at the optimal filtration velocity is shown in Figure 6.

It can be seen from Figure 6 that the counting filtration efficiency of different filter materials increased with an increase in particle size, and the PTFE filter material had the best capture effect. Within the tested particle size range, the effect of the PTFE material was from 0.33% to 16.97% higher than that of the electret material, from 2.20% to 16.12% higher than that of the nanomaterial, and from 1.22% to 8.74% higher than that of the glass fiber material. It can be clearly seen that there were significant differences in the filtration performances among the four filter materials for the filtration of particles smaller than 1.0 μm. This was because, for these particles, Brownian motion plays a dominant role, with small particles continuously moving within the fibers. PTFE had a lower porosity, which increased the particle capture efficiency under the influence of inertia and interception effects. As the particle size increased, the diffusion effect gradually weakened, and the interception and inertial collision effects gradually enhanced [44]. Therefore, for larger particles, their filtration efficiency was not significantly different. However, PTFE was more effective for capturing particles smaller than 1.0 μm.

### 3.5. Changes in Filtration Resistance with Filtration Velocity

**Figure 7:**
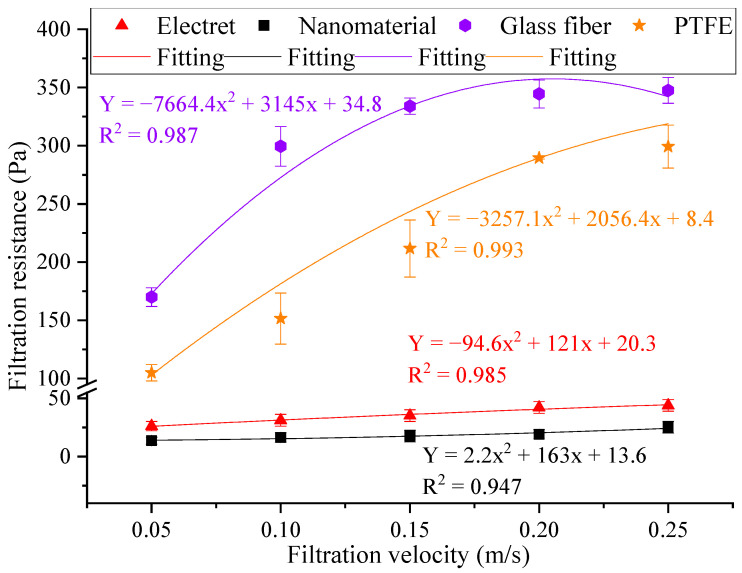
The variation in resistance with different filtration velocities.

The resistance changes in the different materials are shown in Figure 7.

From Figure 7, it can be seen that the resistance and filtration velocity of the different filter materials showed an increasing trend, with a good fitting effect. Within the tested filtration velocity range, the resistance range of the electret material was 26.0–45.0 Pa, the resistance range of the nanomaterial was 13.5–25.5 Pa, the resistance range of the glass fiber material was 166.0–353.0 Pa, and the resistance range of the PTFE material was 101.5–308.5 Pa. The resistance of the filter materials showed the following trend: glass fiber > PTFE > electret > nanomaterial. The main reason for this was that the glass fiber material was relatively thicker, and under the same conditions, the path of airflow through the fibers became longer, which increased the frictional resistance between the particles and the fiber surface. These factors led to a corresponding increase in resistance [45]. In addition, a smaller porosity will affect the uniformity of the airflow velocity field, and the smaller pore size of PTFE materials means that their filtration of particles mainly relies on interception effects, making it easier for pollutants to quickly form a dense filter cake layer on the membrane surface, resulting in a rapid increase in filtration resistance. Therefore, it is also necessary to match the resistance with the filtration efficiency in practical use.

The quality factor value (*QF*) was calculated using Equation (5) [46,47]:

(5) $$QF=\frac{-\ln\left(1-\eta \right)}{\Delta p}$$

where *η* is the filtration efficiency of the filters (%) and ΔP is the filtration resistance (Pa).

Taking the PTFE materials with good results as an example, the quality factors of PM_2.5_ at different velocities were calculated to be 0.00350 Pa^−1^, 0.00348 Pa^−1^, 0.00370 Pa^−1^, 0.00381 Pa^−1^, and 0.00283 Pa^−1^, respectively. When the filtration rate reached 0.2 m/s, the QF value reached its maximum. Therefore, the QF value can be used to measure the comprehensive filtration performances of the air filters. The larger the quality factor, the better the filtering effect, so increasing the quality factor value improved the filtration efficiency.

Overall, in comparing the structures and filtration performances of the four most widely used new filter materials on the market, it was found that PTFE had the best overall performance. However, it still lacked the effective filtration and sterilization of complex microorganisms in the air, as well as the effective adsorption of toxic and harmful gases [48]. It would be useful to explore the development of composite materials that combine the best properties of the different tested filter materials in order to optimize their efficiency and reduce resistance. Therefore, in future research, the development of composite multifunctional materials should be investigated based on the existing research on new materials, which can provide data support for the improvement of indoor air quality.

## 4. Conclusions

In this study, we conducted the testing and analysis of the structural parameters and filtration performances of the four most commonly used new filter materials in the current market; our preliminary conclusions were as follows:

1. The particle size distribution of atmospheric matter in Xi’an was mainly dominated by fine particles. Particles smaller than 2.5 μm accounted for 99.97%, while particles smaller than 1.0 μm accounted for the vast majority (~99.84%), of which particles smaller than 0.5 μm accounted for approximately 69.86%.
2. The four different new filter materials showed a trend of first increasing and then decreasing their filtration efficiency with an increase in the filtration velocity. The filtration efficiency of the materials was as follows: PTFE > glass fiber > nanomaterial > electret. The filtration efficiency of all materials reached its maximum when the filtration velocity was 0.2 m/s. The filtration efficiency of the PTFE material for PM_10_, PM_2.5_, and PM_1.0_ was higher than that of the other three materials, with ranges of 0.87% to 24.93%, 1.21% to 18.69%, and 0.56% to 16.03%, respectively. This was because the PTFE materials had a lower porosity, which had a significant impact on the filtration performance of the filter material.
3. Within the tested particle size range, the effect of the PTFE material was from 0.33% to 16.97% higher than that of the electret material, from 2.20% to 16.12% higher than that of the nanomaterial, and from 1.22% to 8.74% higher than that of the glass fiber material. PTFE was the most effective material for capturing particles smaller than 1.0 μm.
4. Within the tested filtration velocity range, the resistance of the filter material showed the following trend: glass fiber > PTFE > electret > nanomaterial, and the resistance of the four materials showed a good fitting effect. It is also necessary to match the resistance with the filtration efficiency during use.

Overall, PTFE showed the best comprehensive performance, and the focus of subsequent work will be on the effective filtration of microorganisms in the air and the effective adsorption of toxic and harmful gases. This will provide reference data for the research and development of composite multifunctional materials.

## Figures and Tables

**Table 1:** Parameters of the four materials.

Material	Length × Width × Thickness (mm)	Gram Weight (g/m^2^)	Filling Rate (%)	Porosity (%)
Electret	7 × 7 × 0.23	177.55	3.52 ± 0.03	96.48 ± 0.03
Nanomaterial	7 × 7 × 1.22	342.86	2.49 ± 0.02	97.51 ± 0.02
Glass fiber	7 × 7 × 2.65	442.86	2.07 ± 0.03	97.93 ± 0.03
PTFE	7 × 7 × 0.14	169.39	1.65 ± 0.03	98.35 ± 0.03

**Table 2:** Performance differences under different filtration velocities.

Content	Particulate Matter	Filtration Velocity (m/s)				
		0.05	0.1	0.15	0.2	0.25
Electret	PM_10_	27.55	39.90	41.83	52.26	50.38
	PM_2.5_	24.01	37.16	37.69	44.35	41.31
	PM_1.0_	22.48	35.28	36.56	42.69	38.06
Glass fiber	PM_10_	28.57	43.51	47.70	54.52	52.46
	PM_2.5_	28.32	38.17	39.07	46.17	43.70
	PM_1.0_	26.18	34.52	37.53	43.02	40.04
Nanomaterial	PM_10_	43.95	56.62	65.11	69.30	66.36
	PM_2.5_	34.92	41.98	53.09	58.56	54.17
	PM_1.0_	23.97	34.51	46.00	49.51	47.40
PTFE	PM_10_	41.25	59.25	66.77	71.10	67.23
	PM_2.5_	31.61	43.19	56.39	61.43	58.19
	PM_1.0_	28.36	35.83	52.59	55.72	54.69

Note: The average temperature was 21.6 °C~35.3 °C, and the average humidity was 31.3~54.6%.

## Data Availability

The raw data supporting the conclusions of this article will be made available by the authors on request.
